# Epigenetics and Alzheimer’s Disease: A Literature Review

**DOI:** 10.1002/alz.091762

**Published:** 2025-01-03

**Authors:** Bibiana Ruppenthal da Silva

**Affiliations:** ^1^ Universidade do Vale do Rio dos Sinos, São Leopoldo, Rio Grande do Sul Brazil

## Abstract

**Background:**

In recent years, researchers have linked epigenetic factors to numerous diseases, one of them being Alzheimer’s Disease (AD). Those factors may lead to the disease but also serve as a path for new treatments and prevention methods.

**Method:**

A wide selection of articles in the *PubMed* platform that focused on epigenetics, Alzheimer’s Disease, and correlating aspects among them were reviewed.

**Result:**

Previous studies have shown that the main epigenetic factors associated with AD are diet and nutrition, exercise, and stress. An anti‐inflammatory diet rich in copper and zinc results in a decrease in the development of AD since the dysregulation of the nutrients leads to the modification of the amyloid metabolism known to participate in the pathogenesis of AD. At the same time, neurodegeneration may be reduced by up to 45% with the practice of physical exercise, showing that the molecules created by the stimulation could allow discoveries on physiological pathways and non‐drug therapy for prevention. Studies have also found that catecholamine and corticosteroid systems in late‐onset AD can be caused by early life stress. Moreover, epigenetic changes are prone to happen during the prenatal stage and childhood, which indicates the importance of the aspects mentioned in the entire life and not only during the adult years. The absence of the factors mentioned previously could lead to epigenetic mechanisms that modify chromatin and gene expression. These mechanisms are histone acetylation, microRNA, DNA methylation, and histone methylation. The most important ones for AD are the last two that are responsible for silencing gene expression, especially gene *APOE*, crucial for the amyloid β downstream effect on the aggregation of phosphorylated tau in the brain, and when histone H3 is methylated, it affects synaptic transmissions, shaft bursts, and nerve development, and the trimethylation of histone H3 lysine K4 is associated with the expression of genes related to memory and the protein *Zif268* that helps to maintain long‐term potentiation in the recognition memory, respectively.

**Conclusion:**

Overall, epigenetics is a significant area of study to understand the cause of AD and to discover new methods for prevention, diagnosis, treatment, and hopefully one day cure.

